# High Ozone (O_3_) Affects the Fitness Associated with the Microbial Composition and Abundance of Q Biotype *Bemisia tabaci*

**DOI:** 10.3389/fmicb.2016.01593

**Published:** 2016-10-17

**Authors:** Yanyun Hong, Tuyong Yi, Xiaoling Tan, Zihua Zhao, Feng Ge

**Affiliations:** ^1^State Key Laboratory of Integrated Management of Pest Insects and Rodents, Institute of Zoology, Chinese Academy of SciencesBeijing, China; ^2^College of Plant Protection, Hunan Agricultural UniversityChangsha, China; ^3^Department of Entomology, College of Plant Protection, China Agricultural UniversityBeijing, China

**Keywords:** *Bemisia tabaci*, high ozone (hO_3_), fitness, microbial communities, inside of the body, on the surface

## Abstract

Ozone (O_3_) affects the fitness of an insect, such as its development, reproduction and protection against fungal pathogens, but the mechanism by which it does so remains unclear. Here, we compared the fitness (i.e., the growth and development time, reproduction and protection against *Beauveria bassiana* (*B. bassiana*) of Q biotype whiteflies fumigated under hO_3_ (280 ± 20 ppb) and control O_3_ (50 ± 10 ppb) concentrations. Moreover, we determined that gene expression was related to development, reproduction and immunity to *B. bassiana* and examined the abundance and composition of bacteria and fungi inside of the body and on the surface of the Q biotype whitefly. We observed a significantly enhanced number of eggs that were laid by a female, shortened developmental time, prolonged adult lifespan, decreased weight of one eclosion, and reduced immunity to *B. bassiana* in whiteflies under hO_3_, but hO_3_ did not significantly affect the expression of genes related to development, reproduction and immunity. However, hO_3_ obviously changed the composition of the bacterial communities inside of the body and on the surface of the whiteflies, significantly reducing *Rickettsia* and enhancing *Candidatus_Cardinium*. Similarly, hO_3_ significantly enhanced *Thysanophora penicillioides* from the *Trichocomaceae* family and reduced *Dothideomycetes* (at the class level) inside of the body. Furthermore, positive correlations were found between the abundance of *Candidatus_Cardinium* and the female whitefly ratio and the fecundity of a single female, and positive correlations were found between the abundance of *Rickettsia* and the weight of adult whiteflies just after eclosion and immunity to *B. bassiana.* We conclude that hO_3_ enhances whitefly development and reproduction but impairs immunity to *B. bassiana*, and our results also suggest that the changes to the microbial environments inside of the body and on the surface could be crucial factors that alter whitefly fitness under hO_3_.

## Introduction

The global atmospheric concentration of ozone (O_3_) has risen from less than 10 ppb a century ago to 40–60 ppb at present, and it continues to increase at an annual rate of 1∼2% (IPCC, 2013). O_3_ (≥200 ppb) has antimicrobial and deodorizing properties that make it useful in medicine, but as a major air pollutant, it has strong toxic effects on some organisms (Mokoena et al., 2011). The EPA defined O_3_ concentrations ≥350 ppb as hazardous to almost all organisms, and they can affect the survival and distribution of almost all organisms and break down the long-term sustainability of natural ecosystems (Chalfant et al., 2011). In fact, in the forested regions of North America, the concentration of O_3_ reached 200 ppb in Kress et al. (1982), and the maximum atmospheric concentration in Beijing was 273–477 ppb in July 2000 (Wang and Cai, 2002; An et al., 2007), which was far beyond the mean global concentration (40 ppb) and the safe level (100 ppb) defined by the EPA. Insects are both one of the most abundant groups of organisms on the earth and some of the most important members of natural ecosystems, so understanding the effect of hO_3_ on insect fitness is very important.

Elevated O_3_ (eO_3_) (60–80 ppb) affects the fitness of insects, including their growth rate, development time, pupal mass, survival, consumption, feeding behavior, oviposition and protection against fungal pathogens (Jones and Coleman, 1988; Ward and Masters, 2007; Brownlie and Johnson, 2009). The effect of elevated O_3_ on insect fitness can be positive, negative, or neutral (Dermody et al., 2008; Couture and Lindroth, 2012; Capone et al., 2013). To explore the mechanism underlying the effect of eO_3_ on insect fitness, a meta-analysis was conducted, and the results demonstrated that eO_3_ increased fitness. However, neither carbohydrates (starches, sugars, and total non-structural carbohydrates) nor nutrients (N, P, K, Ca, Mn, Mg, and S) responded to eO_3_, whereas the concentrations of terpenes (individual monoterpenes, diterpenes and sesquiterpenes; total resin acids; total monoterpenes; and total terpenes) and phenolics (condensed tannins, hydrolysable tannins, total tannins, flavonoid glycosides, flavonoid aglycones, phenolic acids, and total phenolics), which are generally considered to be protective compounds against insect herbivores, significantly increased by 16 and 8%, respectively. In other words, harmful substances improved insect fitness, and the author explained that O_3_ altered the plant–herbivore interactions, which resulted in changes to the fitness of the herbivores (Valkama et al., 2007). However, apart from the role of plants, the mechanism that determines how insect herbivores adjust themselves to respond to O_3_ is unclear.

Gene *Vg* specifically regulates the synthesis of vitellogenin, which is the precursor of yolk proteins. Yolk proteins are very important for the reproduction of individual insects and the proliferation of insect populations because they provide nutrients for egg development. Insects increase the expression of *Vg* gene to promote individual reproduction and population proliferation, thus improving insect fitness (Shu et al., 2009). Environmental stress including nutritional deprivation, microbial bacterial infection, cutaneous injury, episodic movement, and temperature elevation, affects *Vg* gene expression and modulates insect fitness, while changes in gene expression also help insects mitigate stress (Cervera et al., 2005, 2006; Tauchman et al., 2007; Shu et al., 2009). However, whether hO_3_, as a strong stress factor, affects gene expression and the fitness of insects is unclear.

In addition, microbes are extensively associated with the growth, development and reproduction of their insect hosts and play key roles in their fitness (defense, nutrition and reproduction) (Crotti et al., 2010; Shin et al., 2011; Zucchi et al., 2012; Chu et al., 2016). Environmental microbes negatively affected insect fitness through reducing its population size and fitness (Jacobs et al., 2014). On the other hand, certain environmental microbes also produce antimicrobials that enable insect hosts to repel entomopathogenic bacteria and fungi and to increase their resistance to various parasites, thus improving the fitness of the insect host (Poulsen et al., 2010; Carr et al., 2012; Davis and Engström, 2012; Zucchi et al., 2012). *Wolbachia*, a secondary symbiont of the whitefly, manipulates host reproduction in a variety of ways (inducing parthenogenesis, feminizing genetic males, male-killing, and cytoplasmic incompatibility) (Ishikawa, 2003; Oliver et al., 2003; Gottlieb et al., 2006; Mahadav et al., 2008), and it can increase the fitness of its host by decreasing juvenile development time, increasing the proportion of nymphs that complete development, increasing the adult life span and increasing nutritional supplementation (Xue et al., 2012). Moreover, numerous recent studies have suggested that eO_3_ significantly alters soil microbial community composition (Chung et al., 2006), structure (Kanerva et al., 2008), functional potential/activity (Li et al., 2013), and interaction networks and/or dynamics (Kasurinen et al., 2005; He et al., 2012). However, whether hO_3_ alters the abundance and composition of the microbes associated with insects and, as a result, insect fitness is unclear.

The whitefly, *Bemisia tabaci* (Gennadius) (Hemiptera: Aleyrodidae), is an important agricultural pest worldwide (De Barro et al., 2011) that has caused 100s of millions of dollars of crop damage (Cass et al., 2015). Understanding the effect of hO_3_ on the fitness of the whitefly is very important for predicting the spread of this epidemic and the future protection of the agricultural economy. Kress et al. (1982) screened loblolly pine (*Pinustaeda L*.) with 250 ppb O_3_ and found it to be highly sensitive to O_3_. Clay et al. (2014) exposed infant rhesus macaques (*Macaca mulatta*) to 500 ppb ozone for 8 h/day followed by 9 days of filtered air for 6 months and found that high ozone exposure during postnatal development persistently affects the innate immune response to other environmental challenges. To determine the effect of O_3_ on whitefly fitness, we set our O_3_ treatment concentration to 280 ± 20 ppb.

In this study, our hypothesis is that hO_3_ (280 ± 20 ppb) will alter the fitness of the Q biotype *B. tabaci* via O_3_ affected microbial community composition or O_3_-regulated relative expression of genes associated with development, reproduction and immunity. To test this hypothesis, we determined the effects of hO_3_ on the following characteristics of Q biotype *B. tabaci*: (1) the population parameters of development, reproduction and protection against *B. bassiana*; (2) the expression of genes associated with development, reproduction and immunity to pathogenic fungi; and (3) the community composition and abundance of the microbiota (bacteria and fungi) on the surface and inside of the body.

## Materials and Methods

### Plants and Whiteflies

Tomato seeds were sown in 10 cm diameter plastic pots with commercial peat soil, and the pots were incubated in a screened cage (60 cm × 40 cm × 40 cm) in a greenhouse. The plants were watered every 2 days. The Q-biotype *B. tabaci* were obtained from the Institute of Vegetables and Flowers of the Chinese Academy of Agricultural Sciences (CAAS) on April 22, 2015, and they were identified using the mtDNA *COX*//marker. To conduct the detoxification treatment, Q-biotype *B. tabaci* were fed on an 80 cm-high cotton plant, and after a generation of breeding (20–25 days), they were transferred to the tomato plants. After 3 days, the tomato plants with Q-biotype *B. tabaci* were transported to treatment and control artificial climate chambers incubator at 25 ± 1°C, 70% RH and a 14:10 L:D photoperiod (PRX-450C, Ningbo, Zhejiang, China). Every 20 days, 1–2 fresh tomato plants were added, and the aging plants were removed after 40 days. To avoid error from gene mutations caused by O_3_, the offspring of 2–4 generations of whiteflies were used in the later experiments.

### O_3_ Treatment

In the hO_3_ treatment, O_3_ was formed from the ambient air with an O_3_ generator (3S-A10, Beijing Ligong University, Beijing, China) and then transported to an artificial climate chamber at 25 ± 1°C, 70% RH and a 14:10 L:D photoperiod (PRX-450C, Ningbo, China). To highlight the main effects of ozone and exclude other environmental factors, the O_3_ concentrations were set at 280 ± 20 ppb and monitored in real time (Shenzhen Yiyuntian Electronic CO. LTD). In the treatment, O_3_ was ventilated from 9:00 a.m. to 5:00 p.m, and an artificial climate chamber at 25 ± 1°C, 70% RH and a 14:10 L: D photoperiod (PRX-450C, Ningbo, China) without an O_3_ generator was used as a control.

### Development Time, Fecundity, Adult Longevity and Weight of *B. tabaci*

After a generation of breeding on tomato in both the treatment and control arrangements, 20 pairs of Q-biotype *B. tabaci* adults were randomly selected for transfer to uninfected tomato leaves covered with bags (40 cm × 50 cm, 100X) (treatment and control). The adults were removed after 24 h of infestation, and 30 eggs remained on each leaf. The development time and adult longevity of the *B. tabaci* offspring from egg to adult eclosion and from being newly eclosed to death, respectively, were recorded daily using a 60 × magnifier, and males and females were discerned by the characteristics of the adult shape and recorded. The experiment was repeated three times (randomly selected 30 eggs or individuals per replicate), and 90 data points were collected from both the treatment and control groups. Six pairs of newly eclosed adults from the treatment and control groups were transferred to new leaves, which were also bagged (40 cm × 50 cm, 100X), on six new healthy tomato plants and transferred to a new leaf every 24 h beginning at the base of the plant and moving upward. If a male died, another healthy male from the same treatment was added immediately. The number of eggs from a single female was recorded daily, and the reproduction experiments were repeated five times with 30 data points from both the treatment and control groups. We weighed 10–15 pairs of newly eclosed adults with an electronic balance with a precision of 0.01 mg to obtain an average mass. The weight experiments were repeated fifteen times with 30 data points from both the treatment and control groups.

### Median Lethal Concentrations (LC_50_) of *B. bassiana* and Median Lethal Time (LT_50_) of Exposure in the Whitefly

*Beauveria bassiana* (*B. bassiana*) were cultured on potato dextrose agar (PDA) in Petri dishes (9 cm in diameter) in an incubator (MJ-250, Beijing, China) at 25 ± 0.5°C for 10 days, and conidia were harvested with deionized water containing 0.5% v/v Tween-80 under sterile conditions. The suspensions were shaken on a magnetized stirrer for 20 min to break up the conidial clumps, and the debris was then removed by filtering through four layers of medical gauze. The conidial concentration was estimated with a hemocytometer and then diluted to 10^9^–10^5^ conidia/ml. Conidial viability was determined by culturing the diluted conidial suspension onto PDA at 25 ± 0.5°C for 2 days, and the resulting colony was examined to determine the number of germinating conidia.

To find the median lethal concentrations (LC_50_) of *B. bassiana* against third-instar (pupae) and adult whiteflies, tomato leaves containing a third (≈30) of the instars (pupae) from the treatment and control groups were immersed in the conidial suspensions (10^5^–10^9^ conidia/ml) for 30 s and dried naturally. The leaf petioles were then covered with sterile cotton moistened with sterile water and placed in Petri dishes (9 cm in diameter). Fresh, healthy tomato leaves were treated as above description, and approximately 15 pairs of adult whiteflies (∼24 h eclosion) from the treatment and control groups were transferred to the treated leaves. To avoid the hO_3_ killed conidia, all of the petri dishes were transferred to an artificial climate chamber at 25 ± 1°, 70% RH and a 14:10 L: D photoperiod. Nine replicates (three biological replicates with three technical replicates per biological replicate) for each conidial concentration were performed, and 0.1% v/v Tween-80 was used as a blank control. A third of the instars (pupae) were individually examined under a stereo-zoom binocular microscope at 40X magnification for verification of fungal infection. The mortality data were recorded (twice/day) by counting the dead pupae and adults with fungal spores for 8 days and corrected using Abbott’s formula. The virulence regression equation, the median lethal concentrations (LC_50_) and the median lethal time (LT_50_) values were estimated using DPS software.

### Relative Gene Expression Associated with Development, Reproduction and Immunity to *B. bassiana*

To explore whether changes in gene expression altered whitefly fitness under hO_3_, we determined the mRNA levels of the juvenile hormone (*JH_1_*), vitellogenin (*Vg*), the vitellogenin receptors (*VgR*), the toll-like receptors_1_ (*TLR_1_)*, the toll-like receptor*_7_* genes (*TLR_7_*), *defensin* and the knottin peptides (*knottin*) by real-time quantitative RT-PCR (qRT-PCR). The total RNA of the live whitefly samples from the high O_3_ treatment and the control O_3_ was extracted by TRIzol (Invitrogen) according to the manufacturer’s protocols. The quality and concentration of the purified RNA was assessed based on the ratio of A260/A280 = 1.9∼2.1 and A230/A260 ≥ 2, and the figure generated by an ND-1000 spectrophotometer showed a single peak. The concentrations of RNA were calculated with NanoDrop ND-1000 software and displayed on the attached computer (Nano-Drop Technologies Inc., Wilmington, DE, USA) (Lim et al., 2014). One microgram of total RNA in a 20-μl volume was used for cDNA synthesis with a transcriptor first-strand cDNA synthesis kit (TransGen Biotech, Beijing, China) according to the manufacturer’s protocols, i.e., 1 μg RNA, 4 μl of 5 × buffer, 1 μl RNase inhibitor, 1 μl (20 U/μl) reverse transcriptase, 1 μl (dT)18 with water added to 20 μl; the cDNA was conducted in 55°C for 15 min, at 85°C for 10 s and quickly transferred to ice. To obtain a reliable normalization of the RT-qPCR data, three-fold diluted cDNA templates were used to verify each of the primer pairs to find the optimal concentration range for the qPCR. The optimal cDNA concentration was used in each of the qRT-PCR mixtures (10 μl), and 8 of the optimal cDNA concentrations were randomly selected from the hO_3_ treatment and the control O_3_ and used to validate the stablity of the housekeeping gene (β-*actin*). Real-time detection and analyses were performed using SYBR green dye chemistry with the qPCR kit for SYBR Green I (TransGen Biotech, Beijing, China) and PikoReal2 software (Thermol, USA). The thermal cycling conditions were 95°C for 2 min followed by 40 cycles at 95°C for 15 s and Tm for 30 s, which was followed by a dissociation curve analysis of a ramp from 65 to 95°C with a read every 0.5°C. Nine biological replicates with three technical replicates each were performed. The relative quantification of the mRNA was performed using the Livak-method (2^-ΔΔC_T_^) (Livak and Schmittgen, 2001), and the values obtained for each mRNA were normalized to whitefly β*-actin* (Chen et al., 2015).

### The Composition and Abundance of the Microbial Communities

To explore whether changes in the microbial communities altered whitefly fitness under hO_3_, we determined the composition and abundance of the bacterial and fungal communities from the surface of the whiteflies and inside of the body from the hO_3_ treatment (280 ± 20 ppb) and the control (50 ± 10 ppb) by 16S and 18S sequencing. Approximately 30 live whiteflies were frozen for 3–5 min, soaked in 1 × PBS for 2–3 min, slightly shaken 3–5 times, and centrifuged at low speed for 15 min (repeated three times). The combined supernatants were used to extract the surface microbial genomic DNA using a mini DNA extraction kit (Tiangen, Beijing), and the precipitate was washed three times with 75% (V:V) ethanol and used to extract the genomic DNA from the microbes inside of the body with an insect DNA extraction kit (Mobio, Carlsbad, CA, USA). The quality and concentration of the purified DNA was assessed based on the ratio of A260/A280 = 1.7∼1.9 and A230/A260 ≥ 2, and the figure produced by an ND-1000 spectrophotometer showed a single peak. The concentrations of RNA were calculated with NanoDrop ND-1000 software and displayed on the attached computer (Nano-Drop Technologies Inc., Wilmington, DE, USA) (Zhou et al., 2015). The bacterial universal primers 338 F (5′- ACTCCTACGGGAGGCAGCA-3′) and 806 R (5′-GGACTACHVGGGTWTCTAAT-3′) were used to amplify the V2–V3 region of the bacterial 16S rRNA, and the fungal universal primers 0817 F (5′-GGAAGTAAAAGTCGTAACAAGG -3′) and 1196 R (5′-GCTGCGTTCTTCATCGATGC-3′) were used to amplify the 18S rRNA region of the fungi. The PCR amplifications were conducted in a 20 μL mixture containing 4 μL of 5 × FastPfu buffer, 2 μL of 2.5 mM dNTPs, 0.8 μL of each primer (5 μM), 0.4 μL of FastPfu polymerase, and 10 ng of template DNA, for which the barcode is an eight-base sequence unique to each sample. The PCR reactions were performed in triplicate (95°C for 2 min followed by 25 cycles at 95°C for 30 s, 55°C for 30 s, and 72°C for 30 s with a final extension at 72°C for 5 min). Amplicons were extracted from 2% agarose gels and purified using an AxyPrep DNA Gel Extraction Kit (Axygen Biosciences, Union City, CA, USA) according to the manufacturer’s instructions and quantified using QuantiFluor^TM^-ST (Promega, USA). The purified amplicons were pooled in equimolar and paired-end sequenced on an Illumina MiSeq PE300 platform according to standard protocols. Raw fastq files were demultiplexed and quality-filtered using QIIME (version 1.17) with the following criteria: (i) the 300-bp reads were truncated at any site receiving an average quality score of <20 over a 50-bp sliding window, discarding the truncated reads that were shorter than 50 bp; (ii) exact barcode matching was performed with two nucleotide mismatch in primer matching, and reads containing ambiguous characters were removed; (iii) only sequences that overlapped for more than 10 bp were assembled according to their overlapping sequence. The sequencing reads were assigned to each sample according to the unique barcode of each sample, and pairs of reads from the original DNA fragments were merged using FLASH (Magoc and Salzberg, 2011). Reads that could not be assembled were discarded, and the number of sequences in each sample was greater than 10,000. Operational units (OTUs) were clustered with a 97% similarity cutoff using UPARSE (version 7.1)^1^, and chimeric sequences were identified and removed using UCHIME. The taxonomy of each 16S rRNA gene sequence was analyzed by RDP Classifier^2^ against the SILVA (SSU115) 16S rRNA database using a confidence threshold of 70% (Amato et al., 2013; Quast et al., 2013). The taxonomy of each 18S rRNA gene sequence was analyzed by BLAST software^3^ against the SILVA (LSU123)18S rRNA database using a confidence threshold of 70% (Bimi et al., 2013). Nine replicates were used to analyze the microbial community (bacteria and fungi) on the surface and inside of the body, and 18 bacteria samples and 18 fungi samples from both the treatment and control groups were analyzed. Two 16S libraries and two 18S libraries were constructed in the study because a library accommodated less than 24 samples. To analyze the effect of hO_3_ on the bacteria and fungi from the surface of the whitefly and inside of the body, a Principal Coordinate Analysis (PCoA) was conducted by unweighted unifrac in QIIME.

### Statistical Analyses

**A**n independent-sample t-test was used to analyze the differences in the effects of hO_3_ and control O_3_ on the population parameters, gene expression and the abundance and composition of the microbial community. The level of significance was set at *P* < 0.05, and the fitting agenda was based on the mean value and the standard deviation (SD). These statistical analyses were conducted in SPSS statistics software version 19.0 (IBM, USA). The virulence regression equation, the median lethal concentrations (LC_50_) and the median lethal time (LT_50_) values from the immune bioassay experiment were estimated using DPS software.

To find the link between the macro-level fitness and the micro-level data, we established several regression equations and determined their correlation coefficients. For example, we determined a relationship between the abundance of *Candidatus_Cardinium* and the sex ratio and single female fecundity. We also established a relationship between the development and immunity of the whitefly and the abundance of *Rickettsia*, the weight of newly eclosed adults and the LT_50_.

## Results

### Development Time, Adult Lifespan, Fecundity, Female Ratio and the Weight of Adults Just after Eclosion

**FIGURE 1:**
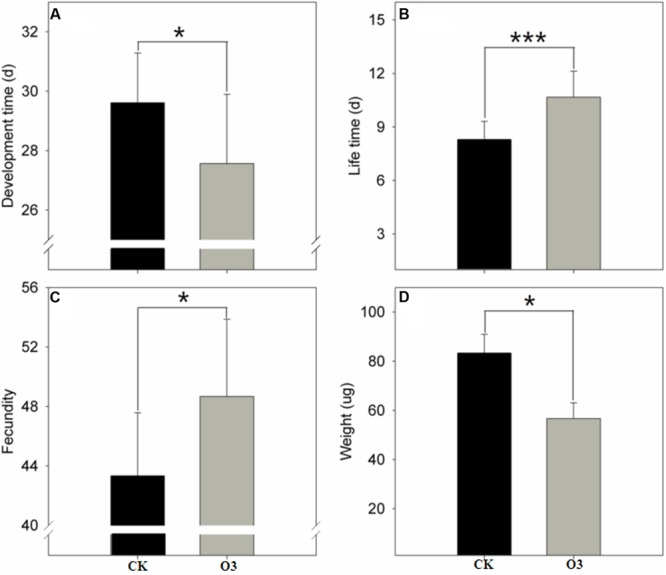
**Whitefly fitness under hO_3_ and control O_3_.** Each value represents the mean value(M) ± standard deviation (SD). ^∗^*P* ≤ 0.05; ^∗∗^*P* ≤ 0.01; ^∗∗∗^*P* ≤ 0.001. **(A)** Is the development time (from egg to adult); **(B)** is the adult life span; **(C)** is the number of eggs from a female; **(D)** is weight of a newly eclosed adult. Gray is hO_3_, and black is control O_3_.

The hO_3_ treatment significantly shortened the total development time from the larval to the adult stages (*t* = -2.06, *df* = 178, *P* = 0.041), prolonged the adult lifespan of the whitefly (*t* = 24.106, *df* = 178, *P* = 0.001), enhanced the number of eggs produced by a female (*t* = 2.051, *df* = 92.249, *P* = 0.043) and the female ratio of the offspring (*t* = -2.001, *df* = 102.249, *P* = 0.048), and decreased the weight of newly eclosed adults (*t* = -2.19, *df* = 178, *P* = 0.030) (**Figure 1**).

### LT_50_ and LC_50_ of Whitefly Exposed to *B. bassiana*

**FIGURE 2:**
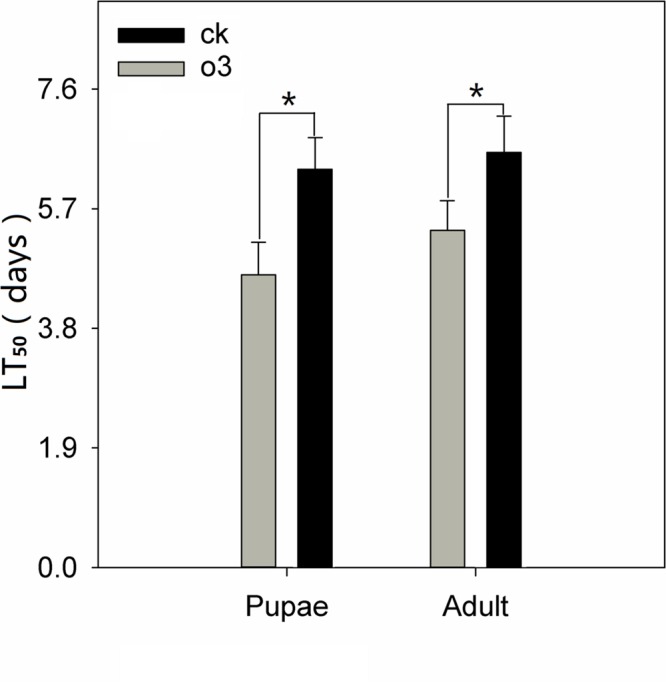
**LT_50_ of whitefly pupae and adults exposed to *B. bassiana* under hO_3_ and control O_3_.** Each value represents the mean value (M) ± standard deviation (SD). Gray is hO_3_, and black is control O_3_ (^∗^*P* ≤ 0.05; ^∗∗^*P* ≤ 0.01; ^∗∗∗^*P* ≤ 0.001).

hO_3_ significantly influenced the immunity of the whitefly pupae and adults to *B. bassiana*; the mortality of the whitefly pupae and adults increased, and the LC_50_ and LT_50_ values decreased and shortened, respectively. The corrected mortality values of the treatment and control groups were 90% at a concentration of 1 × 10^9^ spores⋅mL^-1^ at 8.5 days (treatment pupae) and 10 days (control pupae) (*t* = 2.15, *df* = 16, *P* = 0.047) and 9.33 days (treatment adults) and 10.5 days (control adults) (*t* = 2.14, *df* = 16, *P* = 0.048), respectively. The LC_50_ of the treatment and control groups were 1.97 × 10^5^ (treatment pupae) and 6.95 × 10^7^ (control pupae) and 2.74 × 10^5^(treatment adults) and 8.21 × 10^7^ (control adults) spores⋅mL^-1^; the LT_50_ of the treatment and control groups were 4.67 ± 0.5 days (treatment pupae) and 6.33 ± 0.5 days (control pupae) (*t* = 2.13, *df* = 16, *P* = 0.049) and 5.33 ± 0.5 days (treatment adults) and 6.67 ± 0.5 days (control adults) (*t* = 2.22, *df* = 16, *P* = 0.041) (**Figure 2**; **Table 1**; Supplementary Material).

**Table 1:** The virulence of *Beauveria bassiana* accounting for the variation in whitefly fitness (multiple regressions using backward elimination; α = 0.10 was the criterion for remaining in the model).

	Virulence regression equation	LC50//g/g/g⋅mL^-1^ (95%)	χ^2^	*r*	*R*^2^
O_3_ (pupae)	*Y* = 2.021+1.466x	1.97 × 105 (2.1 × 104 ∼ 6.8 × 105)	0.921	0.975	0.951
CK (pupae)	*Y* = 2.951+1.443x	6.26 × 105 (3.3 × 105 ∼ 7.9 × 106)	1.746	0.986	0.972
O_3_ (adult)	*Y* = 2.122+0.389x	2.74 × 105 (4.5 × 105 ∼ 8.7 × 107)	1.385	0.984	0.968
CK (adult)	*Y* = 1.931+1.360x	7.89 × 106 (0.4 × 106 ∼ 4.7 × 107)	1.436	0.979	0.958

### Expression of Genes Involved in the Immunity, Reproduction and Development of the Whitefly

**FIGURE 3:**
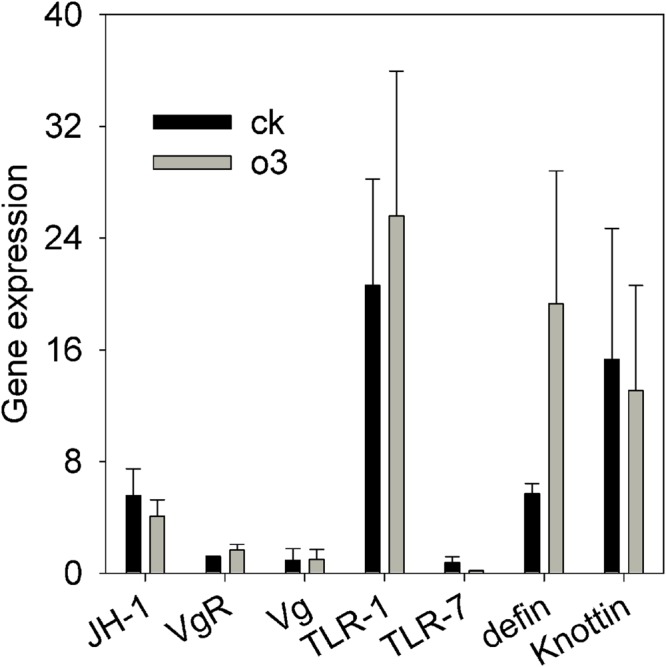
**The relative expression levels of seven whitefly genes associated with development, reproduction and immunity to *B. bassiana* under hO_3_ and control O_3_.** Each value represents the mean value (M) ± standard deviation (SD). *JH-_1_*, juvenile hormone gene; *VgR*, vitellogenin receptor gene; *Vg*, vitellogenin gene; *TLR_1_*, toll-like receptors 1 gene; *TLR_7_*, toll-like receptors 7 gene; *defin, defensin*; knottin; the antimicrobial peptides gene. Gray is hO_3_, and black is control O_3_.

The CT value of the housekeeping gene (β-*actin*) was within the range from 20.75 to 22.13 in the control and hO_3_ groups, which indicated that β-*actin* was stable; that is, it was fit to be the internal control gene in hO_3_. To obtain optimal conditions for real-time qPCR analysis and to ensure the specificity of each primer set, the values of the slope and PCR efficiency (%) for each gene were obtained by a relative standard curve derived from three serial dilutions of pooled cDNA. The qPCR efficiency values for all of the genes were within the range from 90.1 to 102.9 %, and the values of the slope for each gene were within the range from -3.2633 to -3.5839 (Supplementary Material). The ranges of the slope and PCR efficiency values were acceptable for most of the efficiency studies. We found that hO_3_ did not significantly affect the expression of genes involved in immunity, reproduction and development as follows: *t* = 0.653 and *P* = 0.538, *t* = 0.185 and *P* = 0.860 and *t* = 1.464 and *P* = 0.194, for *JH, knottin* and *TLR_7_*, respectively; *t* = -0.053 and *P* = 0.96, *t* = -1.229 and *P* = 0.301, *t* = -0.195 and *P* = 0.852, *t* = -1.005 and *P* = 0.389 for *Vg, VgR, TLR_1_* and *defensin*, respectively (**Figure 3**).

### Significant Changes in the Abundances of the Bacterial and Fungal Communities

**FIGURE 4:**
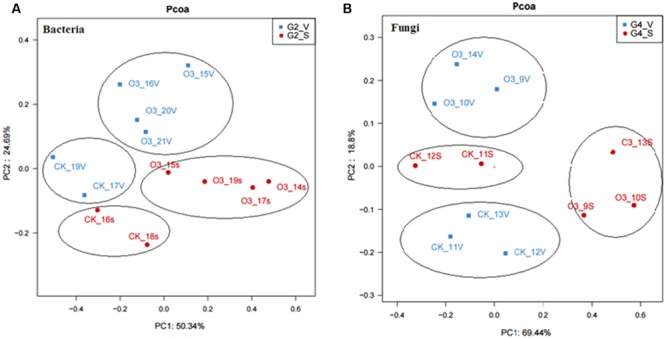
**Principal coordinates analysis (PCoA) of the microbial community based on **(A)** bacteria data and **(B)** fungi data.** CK, control; O_3_, treatment; S, on the surface; V, inside of the body. The values for axes 1 and 2 are the percentages of the variation attributed to the corresponding axes.

**FIGURE 5:**
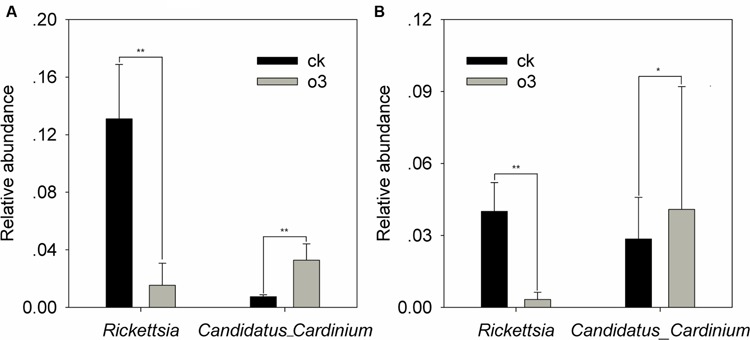
**The differences in the relative abundance of the bacteria on the surface **(A)** and inside of the body **(B)**.** Each value represents the mean value (M) ± standard deviation (SD). Gray is hO_3_, and black is control O_3_. ^∗^*P* ≤ 0.05; ^∗∗^*P* ≤ 0.01; ^∗∗∗^*P* ≤ 0.001. O_3_ significantly decreased the abundance of *Rickettsia* on the surface and inside of the body and increased the abundance of *Candidatus_Cardinium* on the surface and inside of the body.

Samples of the control and hO_3_ bacterial and fungal communities from the surface and inside of the body were well separated from each other, which confirmed that hO_3_ had significant effects on whitefly microbial communities (**Figure 4**). hO_3_ affected the abundance of most bacterial and fungal communities inside of the body and on the surface of the whiteflies, but the abundances of only two bacteria and two fungi were significantly different. hO_3_ significantly decreased the mean abundance of *Rickettsia* up to 11.8% (*t* = 3.907, *df* = 16, *P* = 0.001) on the surface and up to 8.3% (*t* = 3.376, *df* = 12.772, *P* = 0.008) inside of the body and enhanced the mean abundance of *Candidatus_ Cardinium* up to 4.5 times (*t* = 1.843, *df* = 16, *P* = 0.047) on the surface and 1.43 times (*t* = -1.043, *df* = 16, *P* = 0.048) inside of the body (**Figure 5** and Supplementary Material).

**FIGURE 6:**
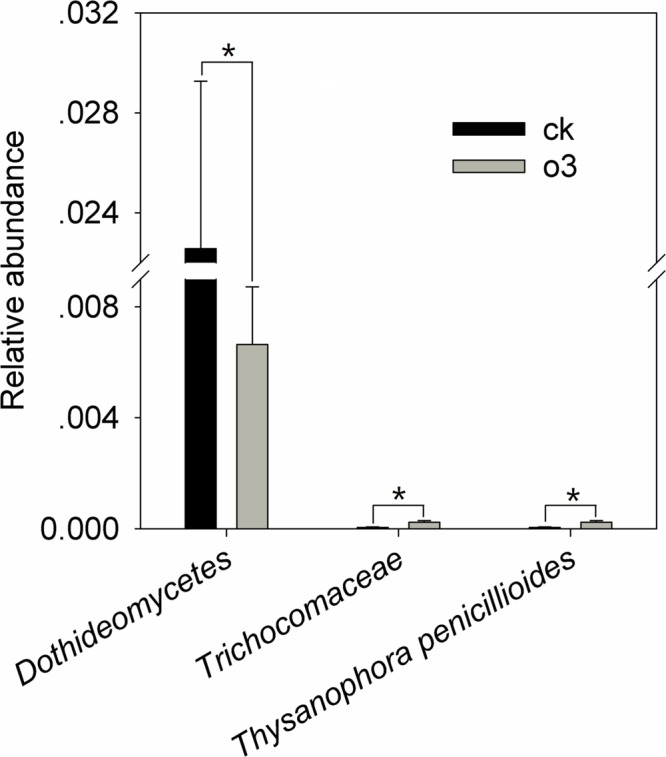
**The differences in the relative abundance of the fungi inside of the body.** Each value represents the mean value (M) ± standard deviation (SD). Gray is hO_3_, and black is control O_3_. ^∗^*P* ≤ 0.05; ^∗∗^*P* ≤ 0.01; ^∗∗∗^*P* ≤ 0.001. O_3_ significantly decreased the abundance of *Dothideomycete*s (class level) and increased the abundance of *Thysanophora penicillioides* (genus level), which belongs to the *Trichocomaceae* (family level).

hO_3_ significantly reduced the mean abundance of *Dothideomycetes* up to 29.4% (*t* = 2.47, *df* = 16, *P* = 0.025) inside of the body at the fungi class level, increased the mean abundance of *Trichocomaceae* 4.4 times (*t* = -2.675, *df* = 11.698, *P* = 0.022) inside of the body at the family level, and increased the mean abundance of *Thysanophora penicillioides* 4.4 times (*t* = -2.675, *df* = 11.698, *P* = 0.022) inside of the body at the fungi genus level (**Figure 6** and Supplementary Material).

### Linear Relationship between Microbial Abundance and Whitefly Fitness

Positive correlations were observed between the abundance of *Candidatus_ Cardinium* and the female ratio and the number of eggs from a female as well as between *Rickettsia* abundance and the weight of newly eclosed adult whiteflies and the resistance to *B. bassiana* (**Table 2**).

**Table 2:** The abundance of *Candidatus_Cardinium* accounting for the variation in the female ratio (X_1_) out of individuals and the number of eggs from a female (X_2_).

Bacteria		Regression equation	*r*	*R*^2^
*Candidatus_Cardinium*	O_3_	*y* = 67.743 X_1_ + 0.00414	0.564	0.318
	CK	*y* = 45.096 X_1_ + 0.00467	0.622	0.387
	O_3_	*y* = 53.613 X_2_ + 0.00414	0.492	0.242
	CK	*y* = 47.071 X_2_ + 0.00467	0.508	0.258
*Rickettsia*	O_3_	*y* = 28.684 X_3_ + 0.00052	0.732	0.536
	CK	*y* = 45.216 X_3_ + 0.00046	0.617	0.381
	O_3_	*y* = 39.743 X_4_ + 0.00434	0.689	0.475
	CK	*y* = 41.087 X_4_ + 0.00467	0.716	0.512

*The abundance of *Rickettsia* accounting for the variation in the LC50 of individuals (X_3_) and the weight of newly eclosed adult whiteflies (X_4_) (multiple regressions using 680 backward eliminations; α = 0.10 was the criterion for remaining in the model; r, correlation coefficient).*

## Discussion

### hO_3_ Affected Whitefly Fitness

As an important agricultural pest worldwide, whiteflies cause severe economic losses every year through direct feeding, excreting honeydew and transmitting plant viruses (De Barro et al., 2011). The whitefly is well known to have a high population growth rate and a remarkable adaptability to environmental stresses, and our previous study showed that eO_3_ (60–80 ppb) decreased the fitness of whiteflies by prolonging their developmental period and enhancing the fitness of *Encarsia formosa* (Cui et al., 2012, 2014). However, in this study, hO_3_ (280 ± 20 ppb) significantly shortened the development time, prolonged the adult lifespan, enhanced the number of eggs produced by a female and improved the fitness of the whitefly. Why are the results of the two studies exactly opposite? It is because the fitness of an insect is affected by many factors, including the duration of exposure to stress, stress intensity (i.e., O_3_ concentration) and the type of exposure [i.e., an indoor growth chamber (IGC) or an open-top chamber (OTC)] (Valkama et al., 2007). In a previous study, the intensity of the eO_3_ treatment (60–80 ppb) was smaller than that of hO_3_ (280 ± 20 ppb), and the eO_3_ exposure time (F_0_–F_1_ generation) was much shorter than the hO_3_ exposure time (F_2_–F_4_ generation). In this study, the type of exposure was through OTCs, where more environmental factors (i.e., CO_2_, O_2_, raining, humidity and temperature) could have a collective effect on whitefly fitness compared to the eO_3_ in an IGC. These differences may reveal a distinct mechanism underlying the effects of O_3_ on whitefly fitness. Moreover, hO_3_ significantly decreased the weight of newly eclosed adult whiteflies, significantly reducing the immunity of whitefly pupae and adults to *B. bassiana*. This result confirmed that the immature stage was less immune to pathogenic fungi (Valkama et al., 2007), suggesting that hO_3_ enhanced whitefly development and reproduction but impaired the protection against *B. bassiana.*

### Relationship between Whitefly Fitness and Relative Gene Expression under hO_3_

It is well known that genes regulate insect fitness. For example, juvenile hormone plays a role in regulating larval development and adult reproduction (Tauchman et al., 2007), and the juvenile hormone gene (*JH-*1) is involved in synthesizing juvenile hormone. Therefore, *JH-*1 regulates insect development. Similarly, *Vg* and *VgR* affect insect reproduction (Cheng et al., 2013), and toll-like receptors-1 (*TLR1)*, toll-like receptors-7 (*TLR7), knottin* and *defensin* affect an insect’s adaptability to its environment and its immunity to pathogenic fungi (Bao et al., 2013; Chen et al., 2015). However, stress also affects insect fitness and is a process that threatens any delicately balanced homeostatic mechanism (Charmandari et al., 2005). Organisms, in turn, make an effort to cope with threats and re-establish homeostasis via stress response systems (Tauchman et al., 2007). For example, Pb and Cd inhibit *Vg* and *VgR* expression, thus decreasing the number of eggs produced and increasing development time (Cervera et al., 2005, 2006; Shu et al., 2009). Heat stress was shown to dramatically decrease the viability of juvenile hormone-deficient *Drosophila melanogaster* mutant *apterous*^56f^, meaning that the *JH* gene is involved in the stress response (Gruntenko et al., 2003). After exposing *Manduca sexta* to various stressors, including nutritional deprivation, microbial infection, cutaneous injury, episodic movement, and elevated temperature, the levels of mRNA expression of the hemolymph juvenile hormone binding protein (*hJHBP*) were less than 50% of those of the control insects (Tauchman et al., 2007). Liu et al. (2014b) and Chen et al. (2015) found a positive correlation between *defensin*, knottin and *TLR*_7_ and whitefly immunity to *B. bassiana*. In the study, hO_3_ significantly shortened development time, prolonged the adult lifespan and increased the number of eggs laid by female, thus increasing whitefly fitness. Moreover, hO_3_ significantly increased the mortality of whitefly nymphs and adults exposed to *B. bassiana* and decreased and shortened the LC_50_ and LT_50_ values, thus significantly decreasing whitefly immunity to *B. bassiana.* However, hO_3_ did not significantly affect the expression of genes associated with development, reproduction and immunity. These results indicate that high ozone altered the fitness and immunity of the whitefly but did not affect the expression of the associated genes.

### Relationship between the Effects of hO_3_ on Whitefly Fitness and Fungal Abundance

hO_3_ significantly reduced the mean abundance of *Dothideomycetes* inside of the body at the fungi class level and significantly increased the mean abundance of *Thysanophora penicillioides*, which belongs to the *Trichocomaceae*, by 4.4 times inside of the body. *Thysanophora penicillioides* is a saprophyte that prevents the development of *Lophodermium* sporophores, which is a pathogenic fungi of pine (Příhoda, 1987), and inhibits the human rhinovirus 3C-protease through the activity of staurosporine (Singh et al., 1991). Due to the lack of information about the function of *Thysanophora penicillioides* on insects, we inferred that the fungus might be involved in the regulation of immune gene expression based on the consistent trend in the abundance of *Thysanophora penicillioides* and the expression of *defensin* and *TLR_1_* and that the fungus secretes the antibiotic staurosporine in humans. The 18S rRNA gene libraries were insufficient and 85.3 and 91.7% of the inside of the body and *in vitro* fungi were unclassified at the family level, so the function of *Dothideomycetes* on the whitefly could not be determined. At the inside of the body fungi genus level, excluding 85.3% of the unclassified fungi, *Thysanophora penicillioides* was dominant, so hO_3_ affected the abundance of dominant fungi inside of the body. However, the potential role of this fungus in the whitefly should be further explored.

### Relationship between the Effect of hO_3_ on Whitefly Fitness and Bacterial Abundance

The whitefly has been shown to harbor an obligatory endosymbiont, *Portiera aleyrodidarum*, and six different facultative symbionts including *Hamiltonella, Arsenophonus, Candidatus_Cardinium, Fritschea, Wolbachia*, and *Rickettsia* (Rao et al., 2015). In this study, the Q-biotype harbored the obligatory endosymbiont, *Portiera aleyrodidarum*, and three different facultative symbionts including *Hamiltonella, Candidatus_Cardinium* and *Rickettsia*, and hO_3_ significantly altered the abundance of *Rickettsia* and *Candidatus_Cardinium*. An earlier study showed that *Rickettsia* improved the adaptation of the whitefly to abnormal environmental conditions (abnormally low temperature) and its resistance to a pathogenic bacterium (*P. syringae*) (Hendry et al., 2014; Su et al., 2014). In this study, the abundance of *Rickettsia* was positively correlated with whitefly weight (adults just after eclosion) and the LT_50_ against *Beauveria bassiana*, supporting the hypothesis that *Rickettsia* are involved in regulating insect growth and immunity to pathogenic fungi (Hendry et al., 2014; Liu et al., 2014b). Additionally, hO_3_ significantly increased the fecundity and female ratio of the Q-biotype whitefly and significantly increased the abundance of *Candidatus_Cardinium* on the whitefly surface and inside of the body up to 4.5 and 1.43 times, respectively. A regression equation showed positive correlations between the female ratio and the number of eggs produced by a female and the abundance of *Candidatus_Cardinium*, which suggests that this symbiont contributes to the regulation of whitefly reproduction by parthenogenesis and feminization. Furthermore, these results are consistent with the fact that *Candidatus_Cardinium* affects host reproductive fitness but does not have an overt effect on the survival (immunity) and size (growth) of adult *Encarsia* (Morag et al., 2013). The existence of a strong correlation between the effect of hO_3_ on fitness and the abundance of bacteria favors a working hypothesis that the changes in the microbial environments inside of the body and on the surface of the whitefly could be a crucial factor underlying changes in fitness under hO_3_. Future experiments should transfer both F_3_ generation whiteflies fed under hO_3_ and CK to the opposite treatment (hO_3_ or CK) to further verify the functions of *Candidatus_Cardinium* and *Rickettsia.*

Symbionts cannot typically be cultivated outside of their hosts, but we detected *Candidatus_Cardinium* and *Rickettsia* in the surface microbial communities. We suspected that the symbiotic bacteria could move inside of the body and *in vitro* of their insect host via the spiracle. Moreover, *Hamiltonella* shared bacteriocytes with *Portiera aleyrodidarum* (Brownlie et al., 2009), and *Rickettsia* appeared in the digestive, salivary, and reproductive organs (testicles and spermatheca), and in the hemolymph, specifically in exceptionally large amounts around bacteriocytes and in fat bodies (Shan et al., 2014). *Candidatus_Cardinium* was also found in different whitefly tissues (Gottlieb et al., 2008). Therefore, our results suggested that hO_3_ increased bacterial abundance and caused a scattered distribution of the bacterial communities.

### Possible Mechanism Underlying the Effects of hO_3_

Previous studies showed that various stressor events stimulated insects to biosynthesize and accumulate juvenile hormone (JH), which impacted the development and survival of the insect. For example, Riddiford (1980) and Cymborowski et al. (1982) proved that stress acted through the endocrine system, and Jones et al. (1980) and Bhaskaran (1981) found that starvation decreased hemolymph trehalose levels and stimulated juvenile hormone (JH) biosynthesis in *Manduca sexta*, increasing the levels of JH and causing the larvae to undergo a highly abnormal sixth stadium. After the fourth stadium, *Manduca sexta* was exposed to various stressors, including nutritional deprivation, microbial infection, cutaneous injury, episodic movement, and temperature elevation, that significantly reduced the levels of hemolymph juvenile hormone binding protein (hJHBP), which transports juvenile hormone to target tissues. This increased JH bioavailability at the target site and thereby impacted the development and survival of the insect (Tauchman et al., 2007). As a strong stressor, hO_3_ stimulates the whitefly to biosynthesize and accumulate JH; in fact, the JH level of the whitefly in this study may be low because development was shortened. What happened to the extra JH? Our hypothesis is that the bacteria and fungi, which increased significantly, consumed the extra JH as part of their ecological function, which includes the regulation of reproduction and development. This hypothesis is supported by other studies. Zheng et al. (2011a,b) found that *Wolbachia* induced the regulation of reproduction in *Drosophila* via the JH pathway, and Liu et al. (2014a) found that *Wolbachia* induced paternal defects in *Drosophila*, probably through an interaction with the JH pathway via the JH response genes *JhI_*26 (the juvenile hormone inducible protein 26 gene) and *CG10433* [the male accessory gland protein (Acp) gene]. *Candidatus_Cardinium* had the same effect as *Wolbachia* in manipulating host reproduction, such as through feminizing genetic males, parthenogenesis, male killing and CI. Therefore, we assumed that *Candidatus_Cardinium* also induced reproduction-regulation in the whitefly via the JH pathway and largely consumed the juvenile hormones, resulting in low JH levels and shortened whitefly development.

## Author Contributions

All authors listed, have made substantial, direct and intellectual contribution to the work, and approved it for publication. That is, FG conceived and designed the experiments; YH performed the experiments, drafted and revised the manuscript; TY revised the manuscript and approved the final version, XT analyzed the data and constructed discussion, and ZZ took part in analyzing and drawing graphs.

## Conflict of Interest Statement

The authors declare that the research was conducted in the absence of any commercial or financial relationships that could be construed as a potential conflict of interest.

## Footnotes

1 http://drive5.com/uparse/

2 http://rdp.cme.msu.edu/

3 http://ftp.ncbi.nlm.nih.gov/blast/executables/blast+/LATEST
