# Supplementary material for: High Ozone (O3) Affects the Fitness Associated with the Microbial Composition and Abundance of Q Biotype Bemisia tabaci
Source: Front Microbiol. 2016 Oct 17;7:1593. doi: 10.3389/fmicb.2016.01593 (PMC5065991; doi:10.3389/fmicb.2016.01593)
Supplement: Supplementary file 1 [file Data_Sheet_1.pdf]

Supplementary Material -1

Independent Samples Test (fitness of whitefly ）

		Equality of Variances		t-test for Equality of Means						
									95% Confidence Interval of the Difference	
		F	Sig.	t	df	Sig. (2-tailed)	Mean Difference	Std. Error Difference	Lower	Upper
adult lifespan	Equal variances assumed	93.029	0.136	24.106	178	0.00	0.111111111	0.136276476	0.384569866	0.162347643
	Equal variances not assumed			24.106	177.681	0.00	0.111111111	0.136276476	0.384577431	0.162355208
reproduction	Equal variances assumed	47.118	0.034	1.99	178	0.048	3.777777778	0.830908152	5.445116966	2.110438589
	Equal variances not assumed			2.051	92.249	0.043	3.777777778	0.830908152	5.447746791	2.107808765
developmental time	Equal variances assumed	75.541	0.103	-2.06	178	0.041	2.259259259	0.629378197	0.996319511	3.522199007
	Equal variances not assumed			-2.102	176.89	0.037	2.259259259	0.629378197	0.994479892	3.524038626
weight	Equal variances assumed	13.999	0.17	2.19	178	0.03	0.888888889	0.458353118	1.808641708	0.03086393
	Equal variances not assumed			2.216	177.612	0.028	0.888888889	0.458353118	1.809603024	0.031825246
female ratio	Equal variances assumed	63.218	0.042	1.96	178	0.052	0.562256209	0.653702073	0.328765324	0.200004477
	Equal variances not assumed			-2.001	102.249	0.048	0.562256209	0.697904779	0.010903379	0.221485027

Supplementary Material -2

Independent Samples Test on gene from whitefly

		Levene's Test for Equality of Variances		t-test for Equality of Means						
									95% Confidence Interval of the Difference	
		F	Sig.	t	df	Sig. (2-tailed)	Mean Difference	Std. Error Difference	Lower	Upper
JH-1	Equal variances assumed	0.976	0.361	0.653	16	0.538	1.4799053	2.264619	-4.0614179	7.021228
	Equal variances not assumed			0.653	14.999	0.542	1.4799053	2.264619	-4.3419607	7.301771
VGR	Equal variances assumed	6.617	0.042	-1.229	16	0.265	-0.5042548	0.410384	-1.5084294	0.499920
	Equal variances not assumed			-1.229	13.224	0.301	-0.5042548	0.410384	-1.7603343	0.751825
TLR-1	Equal variances assumed	3.847	0.098	-0.195	16	0.852	-4.9812776	25.527875	-67.4457369	57.483182
	Equal variances not assumed			-0.195	13.581	0.856	-4.9812776	25.527875	-79.2517856	69.289230
TLR-7	Equal variances assumed	6.325	0.046	1.464	16	0.194	0.6103993	0.417065	-0.4101227	1.630921
	Equal variances not assumed			1.464	13.078	0.237	0.6103993	0.417065	-0.6981274	1.918926
VG-2	Equal variances assumed	0.089	0.776	-0.053	16	0.96	-0.0585834	1.109835	-2.7742515	2.657085
	Equal variances not assumed			-0.053	15.896	0.96	-0.0585834	1.109835	-2.7859437	2.668777
defin	Equal variances assumed	7.259	0.036	-1.005	16	0.354	-13.5953900	13.530593	-46.7035586	19.512779
	Equal variances not assumed			-1.005	13.017	0.389	-13.5953900	13.530593	-56.5181649	29.327385
Knottin	Equal variances assumed	0.2	0.671	0.185	16	0.86	2.2225331	12.040661	-27.2399039	31.684970
	Equal variances not assumed			0.185	15.732	0.86	2.2225331	12.040661	-27.5777102	32.022776

Supplementary Material -3

Independent Samples Test (bacteria on surface of whitefly)

		Equality of Variances		t-test for Equality of Means						
									95% Confidence Interval of the Difference	
		F	Sig.	t	df	Sig. (2-tailed)	Mean Difference	Std. Error Difference	Lower	Upper
Rickettsia, 0.48	Equal variances assumed	11.996	0.143	3.907	16	0.001	0.013916258	0.003561678	0.006365838	0.021466678
	Equal variances not assumed			4.076	15.719	0.001	0.013916258	0.003414399	0.006667544	0.021164972
Candidatus_Cardinium,0.76	Equal variances assumed	7.574	0.558	2.15	16	0.047	0.001909002	0.002288342	0.002942066	0.006760071
	Equal variances not assumed			2.295	11.46	0.044	0.001909002	0.002402594	0.003353293	0.007171298
Acidimicrobiaceae_norank	Equal variances assumed	4	0.063	-0.889	16	0.387	-0.001	4	0.063	0.889
	Equal variances not assumed			-1	9	0.343	-0.001	0.004	0.015	1
Candidatus_Hamiltonella, 0.8	Equal variances assumed	0.358	0.558	0.834	16	0.416	0.001909002	0.002288342	0.002942066	0.006760071
	Equal variances not assumed			0.795	11.46	0.443	0.001909002	0.002402594	0.003353293	0.007171298
Candidatus_Portiera,0.8	Equal variances assumed	0.358	0.558	0.834	16	0.416	0.001909002	0.002288342	0.002942066	0.006760071
	Equal variances not assumed			0.795	11.46	0.443	0.001909002	0.002402594	0.003353293	0.007171298

Supplementary Material -4

Independent Samples Test (bacteria inside of the body of whitefly)

		Equality Levene's Test for Equality of Variances		t-test for Equality of Means						
									Difference	
		F	Sig.	t	df	Sig. (2-tailed)	Mean Difference	Std. Error Difference	Lower	Upper
Candidatus_Portiera	Equal variances assumed	0.006	0.938	0.643	16	0.528	0.05295474	0.082304208	0.119959985	0.225869465
	Equal variances not assumed			0.652	15.857	0.523	0.05295474	0.081251274	0.117845558	0.223755037
Candidatus_Hamiltonella	Equal variances assumed	0.345	0.564	-0.731	16	0.474	0.019767107	0.02703558	0.076566753	0.037032539
	Equal variances not assumed			-0.743	15.948	0.467	0.019767107	0.026592642	0.075647822	0.036113608
Rickettsia	Equal variances assumed	13.002	0.002	3.711	16	0.002	0.040000000	0.011000000	0.018000000	0.063000000
	Equal variances not assumed			3.376	12.772	0.008	0.040000000	0.012000000	0.013000000	0.068000000
Candidatus_Cardinium	Equal variances assumed	4.708	0.07	-2.141	16	0.048	0.017747755	0.017023506	0.053512815	0.018017305
	Equal variances not assumed			-2.222	12.986	0.046	0.017747755	0.015693373	0.051654901	0.01615939

Supplementary Material -5

Independent Samples Test (fungi inside of the body of whitefly)

		Levene's Test for Equality of Variances		t-test for Equality of Means					95% Confidence Interval of the Difference	
		F	Sig.	t	df	Sig. (2-tailed)	Mean Difference	Std. Error Difference	Lower	Upper
Dothideomycetes	Equal variances assumed	3.722	0.078	2.47	16	0.025	0.015918081	0.002378112	0.002378112	0.02945805
	Equal variances assumed			2.269	9.964	0.064	0.015918081	0.006214379	0.00127659	0.033112752
Trichocomaceae	Equal variances not assumed	19.177	0.001	-2.477	16	0.025	0.000183233	0.007016781	0.00127659	0.033112752
	Equal variances assumed			-2.675	11.698	0.022	0.000183233	7.39664E-05	0.000344392	2.20737E-05
Thysanophora penicillioides	Equal variances not assumed	19.177	0.001	-2.477	16	0.025	0.000183233	6.412E-05	0.00033211	3.43559E-05
	Equal variances assumed			-2.675	11.698	0.022	0.000183233	7.39664E-05	0.000344392	2.20737E-05
